# The mosquito effect: regulatory and effector T cells acquire cytoplasmic material from tumor cells through intercellular transfer

**DOI:** 10.3389/fimmu.2023.1272918

**Published:** 2023-12-20

**Authors:** Kaito A. Hioki, Daniel J. Ryan, Iris Thesmar, Adam C. Lynch, Leonid A. Pobezinsky, Elena L. Pobezinskaya

**Affiliations:** ^1^ Department of Veterinary and Animal Science, University of Massachusetts, Amherst, MA, United States; ^2^ UMass Biotech Training Program (BTP), University of Massachusetts, Amherst, MA, United States

**Keywords:** intercellular transfer, tumor, Tregs, CD8 T cells, cytoplasm, exhaustion

## Abstract

The phenomenon of intercellular transfer of cellular material, including membranes, cytoplasm, and even organelles, has been observed for decades. The functional impact and molecular mechanisms of such transfer in the immune system remain largely elusive due to the absence of a robust *in vivo* model. Here, we introduce a new tumor mouse model, where tumor cells express the soluble ultra-bright fluorescent protein ZsGreen, which allows detection and measurement of intercellular transfer of cytoplasm from tumor cells to infiltrating immune cells. We found that in addition to various types of myeloid lineage cells, a large fraction of T regulatory cells and effector CD8 T cells acquire tumor material. Based on the distribution of tumor-derived ZsGreen, the majority of T cells integrate captured cytoplasm into their own, while most myeloid cells store tumor material in granules. Furthermore, scRNA-seq analysis revealed significant alterations in transcriptomes of T cells that acquired tumor cell cytoplasm, suggesting potential impact on T cell function. We identified that the participation of T cells in intercellular transfer requires cell-cell contact and is strictly dependent on the activation status of T lymphocytes. Finally, we propose to name the described phenomenon of intercellular transfer for tumor infiltrating T cells the “mosquito effect”.

## Introduction

The immune system is composed of numerous cell types that either constantly circulate through various tissues or form tissue-resident pools, thereby providing surveillance and protection against possible pathogens as well as contributing to tissue homeostasis. Communication of immune cells between themselves and with non-immune cells is essential for the function of the immune system. In addition to conventional ways of communication through soluble factors or direct cell-cell contact through classical receptor-ligand interactions, many immune cells participate in a very peculiar process of interaction: the intercellular transfer of cellular material. The transfer can occur through trogocytosis, extracellular vesicles or nanotubes (1–6).

Trogocytosis, which is defined as an exchange of membrane components between cells through nibbling, was the first example of material sharing reported in the literature (7–10) and since has likely been the most well-documented transfer phenomenon. It has now been demonstrated that many surface proteins, including major histocompatibility complex (MHC) molecules, costimulatory and adhesion molecules, and inhibitory ligands, can be transferred between various cells (11–16), resulting in different outcomes. For example, the transfer of MHC class I/peptide complexes to dendritic cells, termed cross-dressing, was shown to increase antigen-presentation (17–19). Splenic marginal zone B cells, which are otherwise considered to be poor antigen presenters to T cells, also acquire this function by obtaining peptide/MHC class II complexes from surrounding dendritic cells (20). On the other hand, exchange of natural killer (NK) activating receptors and their ligands between NK cells and target cells was shown to decrease cytotoxicity of NK cells and even lead to NK cell death (21, 22). Likewise, capture of CD80/CD86 molecules from antigen-presenting cells by CTLA-4 on T cells followed by CTLA-4 endocytosis and degradation down-regulates immune responses (23). Interestingly, small GTPases were implicated in the regulation of trogocytosis where T cell receptors (TCR) bound to peptide/MHC complexes are internalized by T cells during immunological synapse formation (24).

Cell-to-cell communication through extracellular vesicles, mostly exosomes, has gained much attention in the last 20 years. Exosomes can transfer bioactive molecules, thereby modulating immune responses (25, 26). Antigen-presenting cells (APCs) can uptake exosomes containing MHC/peptide complexes and present those antigens to T cells (27–29). Transfer of genetic information, including miRNAs, between immune cells via exosomes was first demonstrated by Valadi et al. (30) and later confirmed in many other studies (31–34). T cells were recently shown to elongate their telomeres by accepting telomere-containing vesicles from APCs during synapse formation, and as a result diverting their fate from senescence to long-lived memory (35).

Interestingly, immune cells have been recently shown to utilize intercellular transfer as a mechanism of controlling tissue homeostasis. For example, macrophages can uptake mitochondria from adipocytes, which allows them to regulate metabolic homeostasis in white adipose tissue (36). Acquisition of CD14, a component of the LPS receptor, by human liver CD8 T cells from surrounding myeloid cells, endows CD8 cells with immunomodulatory properties under steady state (37).

Our knowledge of intercellular transfer mostly comes from *in vitro* systems, and those studies are usually focused on specific cell types. In the past decade, more publications appeared that use reliable *in vivo* models to demonstrate intercellular transfer. However, to what extent different immune cells in the same spatiotemporal settings can participate in this process still remains unclear. Therefore, we chose to analyze the tumor microenvironment (TME) in mice as a special niche that consists of diverse immune cell types, with the goal to obtain an unbiased view of the intercellular transfer capabilities among tumor infiltrating lymphocytes (TILs). Here we report that although all immune subsets within the TME acquire tumor material, the degree of transfer varies between cell types. We show that within the lymphoid lineage compartment, antigen-experienced T cells are the most active participants in intercellular transfer of tumor cell cytoplasm. We also demonstrate that T cells which acquired tumor material may have different metabolic status and immune functions compared to T cells that did not participate in intercellular transfer. Since such transfer requires cell-cell interaction, we propose to call this phenomenon the “mosquito effect”.

## Results

### Different immune cell populations acquire tumor material within TME

**Figure 1:**
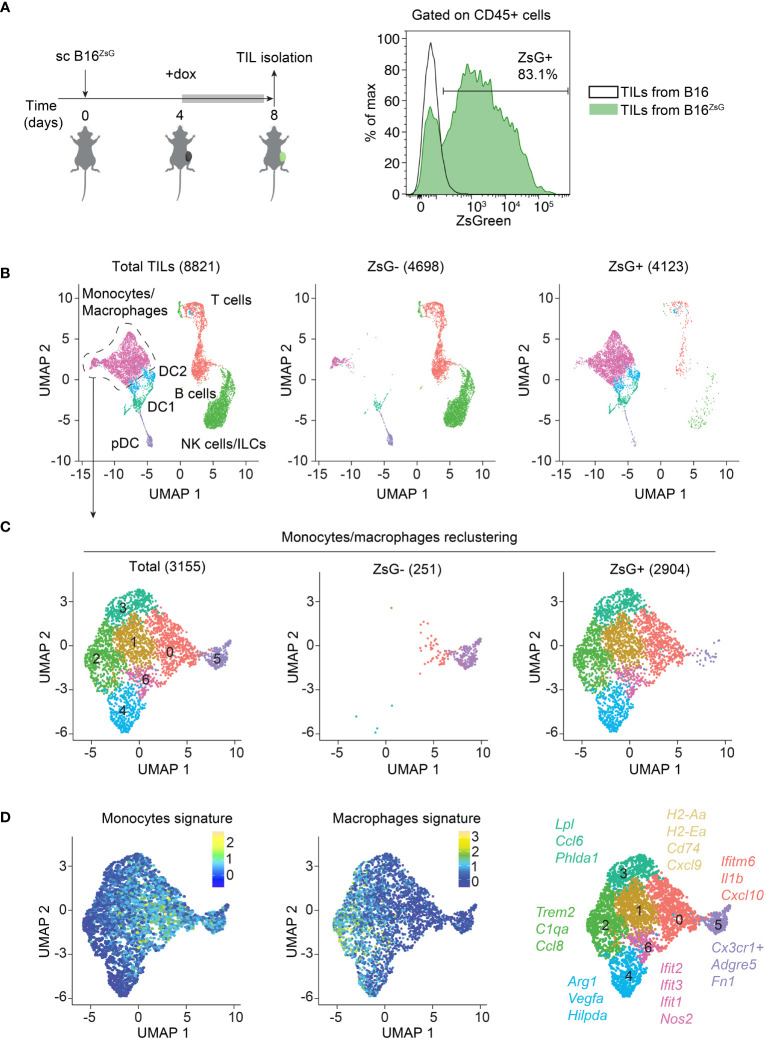
The majority of immune cells acquire tumor material within the TME. **(A)** Experimental design, and representative flow cytometry analysis of ZsG fluorescence in CD45+ TILs isolated from B16 and B16^ZsG^ tumors. **(B–D)** Total CD45+ TIL scRNA-seq data analyses. **(B)** UMAP visualization of scRNA-seq data from total (left), ZsG- (middle) and ZsG+ (right) TILs from B16^ZsG^ tumors. The number of analyzed single cells are indicated in parenthesis. **(C)** UMAP plots of re-clustered monocytes/macrophages. **(D)** UMAP plots of monocyte (left) and macrophage (middle) signatures in re-clustered monocytes/macrophages with key marker genes in each cluster (right).

To assess intercellular transfer from tumor cells to the tumor-infiltrating immune cells *in vivo*, we generated a B16-F10 melanoma cell line that expresses a cytoplasmic ultra-bright fluorescent protein, ZsGreen (ZsG) under the control of doxycycline (dox) (38). Cells were single cell cloned and a B16^ZsG^ clone with the brightest fluorescence was used in subsequent experiments (**Supplementary Figures 1A, B**). B16^ZsG^ cells were subcutaneously (sc) injected into wild-type C57BL/6 mice. Expression of ZsG was induced by giving mice dox-containing water for four days, after which TILs were isolated from tumors on day 8 post-inoculation (**Figure 1A**, left). Examination of TILs by flow cytometry revealed that the majority of CD45-positive cells acquired ZsG-fluorescence from tumors (**Figure 1A**, right). To identify immune cell types that are susceptible to intercellular transfer, we sorted ZsG-negative and ZsG-positive cells from total CD45+ TILs from B16^ZsG^ tumors and performed single-cell RNA sequencing (scRNA-seq) on both populations. Cells were clustered based on the expression of cell type-specific genes (**Supplementary Figure 2A**), then assigned to 7 TIL populations and projected on a UMAP (Uniform Manifold Approximation and Projection) plot (**Figure 1B**). The ZsG-negative sample was enriched in lymphoid lineage cells, while the ZsG-positive sample mostly contained myeloid cells, accounting for more than 90% of the group. Among myeloid lineage cells, the vast majority of monocyte/macrophage-like cells and conventional dendritic cells (DC2 and most of DC1) were ZsG-positive, while plasmacytoid DCs (pDC), a small portion of DC1 cells and a small portion of monocytes/macrophages appeared ZsG-negative. To define monocyte/macrophage subpopulations more accurately, we further re-clustered the monocyte/macrophage group into 7 subsets (**Figure 1C**; **Supplementary Figure 2B**). Cells in clusters 0,1, 5 and 6 were characterized by the expression of monocytic markers, while clusters 2, 3 and 4 expressed a stronger macrophage signature (**Figure 1D**; **Supplementary Figure 2C**). Moreover, macrophage clusters 2-4 were characterized by genes associated with immunosuppression, such as *Trem2*, *Arg1*, and *Vegfa*, while cells in monocyte-like clusters 0,1 and 6 expressed pro-inflammatory genes, including *Ifit1*, *Ifit2*, *Ifit3*, *Nos2*, *Cxcl9*, and *Cxcl10* and genes from the MHC class II presentation pathway, such as *H2-Aa*, *H2-Ea*, *Cd74*, and *H2-DM*. Cluster 5 in the ZsG-negative group had a high expression of *Cx3cr1* (**Supplementary Figure 2D**), which has been previously shown to be expressed by non-classical blood monocytes (39, 40). During homeostasis, Cx3cr1^hi^ monocytes patrol vasculature for damaged endothelial cells; however, during inflammation they can extravasate into tissues. Thus, cluster 5 cells may represent newcomers from blood that have not participated in intercellular transfer yet, and therefore appear to be ZsG-negative. Of note, the low number of lymphocytes, especially T cells, in ZsG-positive group was insufficient for further analysis of lymphoid populations. In sum, with the exception of pDCs, the majority of myeloid cells are capable of acquiring tumor material regardless of their immunosuppressive or pro-inflammatory status.

**Figure 2:**
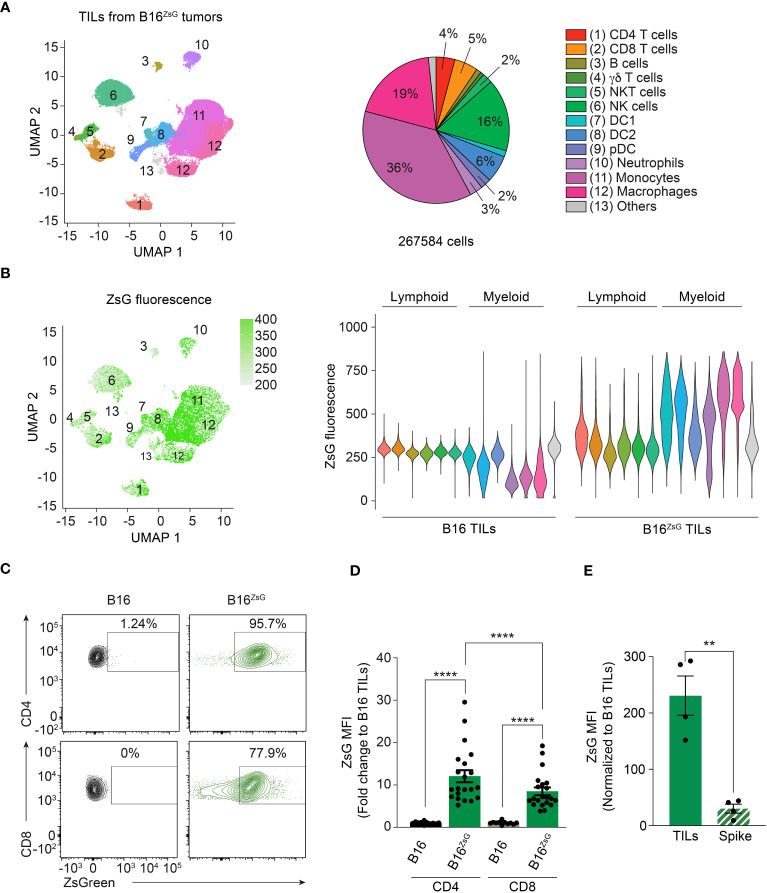
CD4 and CD8 T cells participate in the intracellular transfer. **(A)** UMAP visualization of flow cytometry data with a pie chart representation of TIL population abundance in B16^ZsG^ tumors. **(B)** UMAP plot (left) and violin plots (right) of ZsG intensity in each population from B16 and B16^ZsG^ tumors. **(C)** Representative flow cytometry analysis showing ZsG transfer into CD4 and CD8 T cells from B16^ZsG^ tumors. **(D)** Summary graph of ZsG MFI in CD4 and CD8 TILs from B16^ZsG^ tumors presented as a fold change relative to B16 tumors. **(E)** Summary graph of ZsG MFI in intratumorally injected CD45.1 “spike” CD8 T cells in comparison to endogenous CD45.2 CD8 TILs from B16^ZsG^ tumors. **(D, E)** Mean ± s.e.m, Mann-Whitney U test between B16 TILs and B16^ZsG^ TILs and Wilcoxon test between CD4 and CD8 B16^ZsG^ TILs **(D)**, paired T-test **(E)**: **p<0.01, ****p<0.0001. Data represent three independent experiments **(A–C)** or are pooled from at least three independent experiments **(D, E)**.

To validate the data obtained by scRNA-seq and to compare intercellular transfer efficiency between different cell types including lymphocytes, we analyzed TILs from a B16^ZsG^ tumor by multicolor flow cytometry using the Cytek Aurora instrument. First, we defined 12 populations based on the surface expression of lineage-specific markers (**Supplementary Figure 3A**). We then visualized the distribution of TIL populations on a UMAP plot (**Figure 2A**). Next, we assessed the intensity of ZsG fluorescence among different populations. Projection of ZsG intensity on UMAP clusters and individual comparison of the populations revealed that myeloid cells with phagocytic activity including monocytes, macrophages and conventional DCs (DC1 and DC2) had the highest level of ZsG fluorescence (**Figure 2B**), while pDCs and neutrophils were less bright. Surprisingly, we detected significant acquisition of tumor-derived ZsG within the lymphoid compartment, although with lesser fluorescence intensity than in most myeloid populations. Among the lymphoid lineage cells, CD4 and CD8 T lymphocytes demonstrated the highest ZsG fluorescence, while γδ T cells, NK and NKT cells had medium to low fluorescence, and B cells exhibited the least amount of transfer in B16^ZsG^ tumors (**Figure 2B**). For a better visualization of transfer in T cells the data are shown as contour flow plots (**Figure 2C**). Interestingly, CD4 T cells had significantly higher ZsG-signal than CD8 lymphocytes (**Figures 2B–D**). Similar results were obtained with a MC38^ZsG^ adenocarcinoma tumor model, indicating that the acquisition of tumor material by immune cells is not specific to the B16 melanoma tumor model (**Supplementary Figures 3B, C**). To ensure that intercellular transfer in T cells was not an artifact of sample preparation, we injected intratumorally “spike” CD45.1+ polyclonal CD8 cytotoxic T lymphocytes (CTLs) into B16^ZsG^-bearing congenic CD45.2 mice, immediately harvested tumors and isolated TILs for analysis. We found no evidence of non-specific transfer of ZsG into CD45.1 CTLs in comparison to endogenous CD45.2 CD8 T cells (**Figure 2E**). Similar results were obtained with “spike” injection of activated polyclonal CD4 T cells (**Supplementary Figure 3D**). Overall, our data demonstrate that the vast majority of immune cells have the ability to obtain tumor cytoplasmic material; however, the level of transfer varies between different cell types.

### T cells obtain and integrate cytoplasm from tumor cells

**Figure 3:**
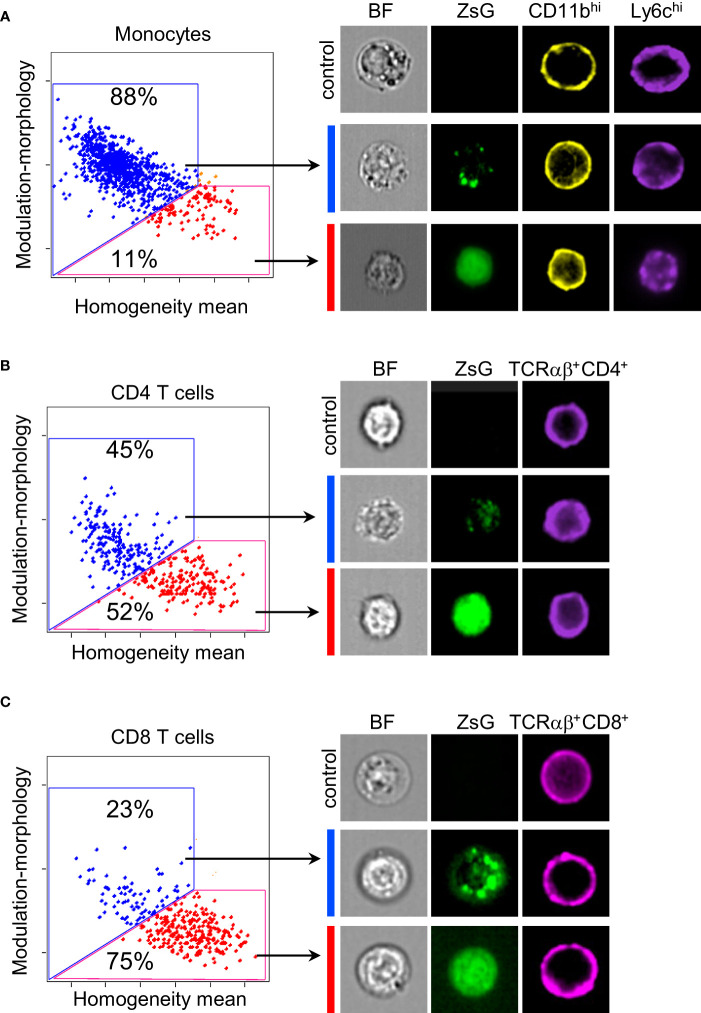
Subcellular distribution of ZsG in TILs. **(A–C)** Quantification of frequency of monocytes **(A)**, CD4 **(B)** and CD8 **(C)** T cells with different cellular localization of ZsG (left panels) and representative images (right panels). Modulation-morphology feature (blue) and homogeneity feature (red) determine granular versus homogeneous distribution. All images were acquired at 60X magnification. Image display gains for ZsGreen were set to the same parameters with the IDEAS software to allow for visual comparison of intensity between images. Control, cells isolated from B16 tumors; BF, bright field.

Myeloid cells are well-known for their phagocytic abilities, therefore, it was not surprising to find them among cells that acquired the most ZsG fluorescence. On the other hand, it is very intriguing that T cells, which are not phagocytes, also quite extensively obtain ZsG from tumor cells. To determine the subcellular localization of ZsG protein in TILs, we examined images of sorted monocytes, as a representative population from the myeloid compartment, and both CD4 and CD8 T lymphocytes using the ImageStream flow cytometer. To ensure that the true intracellular space is analyzed, only cells that had a clear circular pattern of staining for surface markers (CD11b for myeloid cells and CD4/CD8 for T cells) were taken into consideration. Using a combination of the texture features (morphology, modulation and homogeneity) we quantified the cellular localization of ZsG and found that the majority of monocytes (88%) had a punctate pattern of ZsG, which might correspond to phagocytic or endocytic granules (**Figure 3A**). In contrast, both CD4 and CD8 T cells exhibited two patterns of ZsG fluorescence in the cytoplasm: homogeneous and punctate. While approximately equal proportions of CD4 cells had punctate or diffused patterns (**Figure 3B**), more CD8 cells (~75%) demonstrated homogenous distribution of ZsG (**Figure 3C**). Thus, in comparison to myeloid cells that mostly retain acquired tumor material in vesicles, T cells seem to have a mechanism that allows them to incorporate transferred cytoplasm into their own.

### Exhausted CD8 T cells and Tregs are the major T cell populations that acquired tumor material

**Figure 4:**
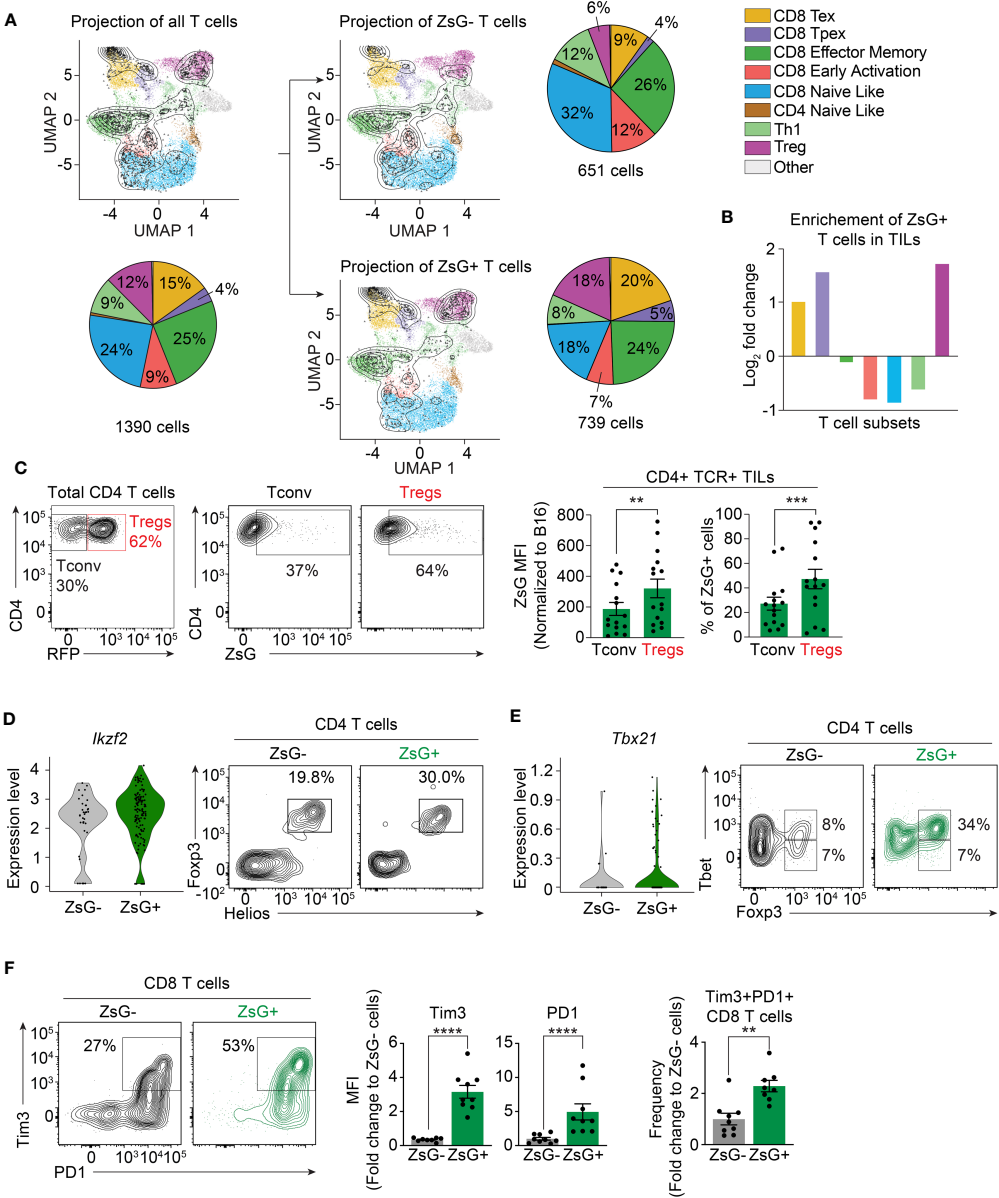
Exhausted CD8 T cells and Tregs are the most susceptible to intercellular transfer. **(A, B)** ProjecTILs analysis of T cell scRNA-seq data. **(A)** Projection of total, ZsG- and ZsG+ T cell scRNA-seq data on the reference atlas with pie charts showing proportions of each TIL subset. **(B)** Fold-change in the frequency of ZsG+ T cells compared to that of ZsG- T cells for each TIL subtype. **(C)** Representative flow cytometry analysis showing ZsG transfer into CD4 conventional TILs compared to Tregs from B16^ZsG^ tumors with summary graphs of ZsG MFI and frequency of these populations. **(D)** Violin plots showing expression level of *Ikzf2* in ZsG- and ZsG+ Tregs (scRNA-seq data) with representative flow cytometry analysis demonstrating a higher percentage of Helios+Foxp3+ cells among ZsG+ CD4 TILs compared to ZsG- CD4 TILs from B16^ZsG^ tumors. **(E)** Violin plots showing expression level of *Tbx21* in ZsG- and ZsG+ Tregs (scRNA-seq data) with representative flow cytometry analysis demonstrating a higher percentage of Tbet+Foxp3+ cells among ZsG+ CD4 TILs compared to ZsG- CD4 TILs from B16^ZsG^ tumors. **(F)** Representative flow cytometry analysis showing Tim3 and PD-1 expression in ZsG- and ZsG+ CD8 TILs from B16^ZsG^ tumors with summary graphs of Tim3 and PD-1 MFI and frequency of the Tim3+PD-1+ population presented as fold changes relative to CD8 TILs from B16 control tumors. **(C, F)** Mean ± s.e.m, paired T-test **(C)**, Mann-Whitney U test **(F)**: **p<0.01, ***p<0.001, ****p<0.0001. Data represent three independent experiments **(C–F)** and are pooled from at least three independent experiments **(C, F)**.

There is great phenotypic and functional heterogeneity among T cell populations in the TME (41). Therefore, we hypothesized that various T cell subsets may have different susceptibility to intercellular transfer. Our previous strategy to sort bright ZsG-positive cells (**Figure 1**) did not allow us to reliably analyze scRNA-seq data for T cells in the lymphoid compartment, as the majority of ZsG-positive lymphocytes fell into the “ZsG-negative” gate, and therefore were excluded from this analysis. To appropriately determine the composition of T cell subsets that are involved in intercellular transfer, we then sorted ZsG-positive and ZsG-negative T cells from B16^ZsG^ tumors and performed scRNA-seq. To identify T cell populations, we took advantage of a previously published reference atlas (ProjecTILs) for T cells isolated from murine B16 tumors (42) (**Supplementary Figure 4A**). Projection of our T cell scRNA-seq data onto this reference atlas demonstrated the presence of 8 previously identified T cell clusters in our samples: exhausted CD8 T cells (CD8-Tex), precursors of exhausted CD8 T cells (CD8-Tpex), CD8 effector-memory cells (CD8-EM), early-activated CD8 cells (CD8-EA), CD4 and CD8 naïve-like clusters (CD4-NL, CD8-NL), T-helper 1 cells (Th1) and regulatory CD4 T cells (Treg) (**Figure 4A**; **Supplementary Figure 4B**). We excluded the CD4-NL cluster from the further quantification analysis because very few cells were detected. The separate projection of ZsG-negative and ZsG-positive data sets revealed that T cell subsets from the two groups mapped differently to the reference populations (**Figure 4A**). Specifically, the ZsG-positive sample had an almost 3-fold enrichment in Tregs, 2.5-fold enrichment in CD8-Tpex and 2-fold enrichment in CD8-Tex cells (**Figure 4B**). In contrast, early-activated CD8 cells, naïve-like T cells and Th1s were underrepresented, while the CD8 effector-memory population had an equal distribution between ZsG-negative and ZsG-positive groups. These data suggest that Tregs and CD8-Tex cells are the main participants in intercellular transfer within the TME.

Next, we confirmed our results using flow cytometry. For Tregs, we used Foxp3^RFP^ reporter mice, in which Foxp3-expressing Tregs are marked by RFP fluorescence. Analysis of TILs isolated from day-8 B16^ZsG^ tumors from Foxp3^RFP^ mice demonstrated that the ZsG MFI in Tregs and the frequency of ZsG-positive Tregs were significantly higher in comparison to conventional CD4 T cells (**Figure 4C**). It has been previously shown that Tregs found in tumors are of thymic origin and thus express the transcription factor Helios, unlike induced peripheral Tregs that are Helios-negative. Our scRNA-seq and flow cytometry data are in agreement with the literature, as both ZsG-negative and ZsG-positive scRNA-seq samples had equal levels of *Ikzf2* (the gene that encodes Helios) expression, and all Foxp3-positive cells among sorted ZsG- and ZsG+ CD4 TILs were Helios-positive (**Figure 4D**). Next, we examined the expression of Tbet in ZsG-negative and ZsG-positive Tregs, the transcription factor that marks the most potent suppressor cells (43), and found that the ZsG-positive group was enriched in Tbet+ Tregs in comparison to ZsG-negative control cells (**Figure 4E**). To validate CD8-Tex cells, we assessed the expression levels of inhibitory receptors PD1 and Tim3 on CD8 T cells from B16^ZsG^ tumors. ZsG-positive CD8 T lymphocytes had a two-fold increase in PD1+Tim3+ double-positive cells in comparison to the ZsG-negative CD8 T cell population (**Figure 4F**). Thus, tumor-infiltrating Tregs and exhausted CD8 T cells appear to be the predominant populations involved in intercellular transfer from tumor cells within the TME.

### Intercellular transfer is dependent on the activation status of T cells and cell-cell interaction

**Figure 5:**
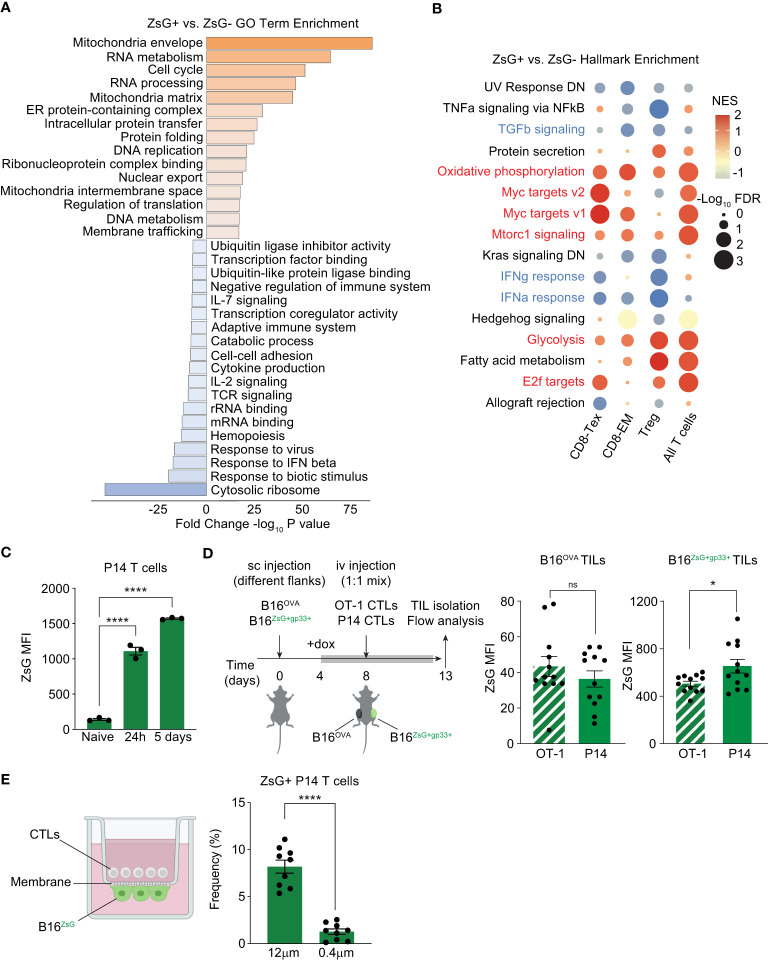
T cell activation status is positively correlated with the degree of intercellular transfer. **(A, B)** Pathway enrichment analyses of T cell scRNA-seq data. **(A)** Metascape gene ontology analysis of all ZsG+ TILs compared to all ZsG- TILs. **(B)** Bubble plot showing enrichment of select hallmark gene sets in ZsG+ TIL subsets compared to ZsG- TIL subsets. Positively enriched pathways are in red, downregulated pathways are in blue. **(C)** ZsG MFI in naïve, activated (by plate-bound antibodies for 24h), or fully differentiated (activated for 5 days) P14 T cells, that were co-cultured with gp33-loaded B16^ZsG^ tumor cells for 24 hours. **(D)** Experimental design and summary graphs of ZsG MFI of OT-1 and P14 CTLs isolated from B16^Ova^ and B16^ZsG+gp33+^ tumors. Data were adjusted to non-fluorescent B16^Ova^ and B16^gp33+^ tumors. **(E)** Experimental design of the transwell assay to coculture P14 CTLs and B16^ZsG^ cells across 12 μm or 0.4 μm pore membranes, and frequency of ZsG+ P14 CTLs collected 24 hours later. NES, normalized enrichment score. **(C–E)** Mean ± s.e.m, unpaired T-test **(C, E)**, paired T-test **(D)**: ns, nonsignificant, *p<0.05, and ****p<0.0001. Data are from three independent experiments **(C–E)**.

To begin to understand the differences between T cells that can acquire tumor material and those that cannot, we compared the differentially expressed genes between all ZsG-negative and all ZsG-positive T cells and searched for enrichment of gene ontology (GO) terms using Metascape (**Figure 5A**). We found that GO terms associated with metabolically active cells (Mitochondria envelope, Cell cycle, RNA and DNA metabolism, Intracellular protein transfer) prevailed in the ZsG-positive group. On the other hand, many GO terms associated with the immune response (Response to IFN beta, TCR signaling, IL-2 signaling, Cytokine production, Adaptive Immune response) were underrepresented, indicating that despite the metabolically active state, T cells that acquire tumor material are less functional. Interestingly, the most negatively enriched GO term in T cells that received tumor material was “Cytosolic ribosome”, pointing to compromised translation in these cells. To further explore the differences between ZsG-negative and ZsG-positive T cells, we performed Gene Set Enrichment Analysis (GSEA) using the 50 Hallmark gene sets. Although several cell types and pathways did not meet our criteria for significance (-Log_10_FDR>1, NES>+/-1), Tregs, CD8-Tex and CD8-EM populations demonstrated that the most differentially enriched pathways in ZsG-positive groups were those associated with T cell activation, such as Myc targets, E2f targets, Oxidative phosphorylation, Glycolysis and Mtorc1 signaling (**Figure 5B**). Of note, cytokine response pathways, including TGF-beta, IFN-gamma and IFN-alpha signaling were downregulated in ZsG-positive cells, which indicates suppressed immune function and is consistent with our Metascape analysis. Overall, these data suggested that activation status of T cells is positively correlating with the degree of intercellular transfer. We confirmed this observation in an *in vitro* experiment, where naïve, 24h-activated or fully differentiated CTLs from P14 TCR transgenic mice were cocultured with B16^ZsG+^ tumor cells loaded with gp33-41 peptide (the cognate peptide from the lymphocytic choriomeningitis virus (LCMV) presented in the context of the D^b^ molecule) and analyzed by flow cytometry. Indeed, more activated T cells exhibited greater ZsG fluorescence (**Figure 5C**), supporting the transcriptomics data and, also, raising the question of whether intercellular transfer is dependent on TCR/MHC interaction and the presence of antigen (Ag). To test this possibility, we used an Ag-specific *in vivo* model where mice were sc injected with fluorescent B16^ZsG+gp33+^ and non-fluorescent B16^OVA+^ tumor cells, expressing chicken ovalbumin (OVA), on separate flanks followed by adoptive transfer of P14 and OT-I CTLs and further analysis of ZsG fluorescence in TILs (**Figure 5D**). Both P14 and OT-I T cells isolated from B16^ZsG+gp33+^ tumor exhibited green fluorescence, with Ag-specific P14 T cells being brighter than Ag-nonspecific OT-I T cells (**Figure 5D**, left graph). To our surprise, both types of T cells isolated from non-fluorescent B16^OVA+^ tumor from the opposite flank also acquired ZsG, although to a much lesser extent than T cells from the fluorescent tumor (**Figure 5D**, right graph). These data suggest that: (1) Ag-stimulated T cells are the most sensitive to the intercellular transfer, while the expression of cognate Ag on tumor cells is not required for this process, and (2) It is possible that the intercellular transfer occurs through extracellular vesicles (EV).

To determine whether EVs play any role in intercellular transfer, we performed a transwell assay with some modifications. Two types of inserts with a pore size of 0.4 μm and 12 μm were used in the assay. B16^ZsG^ tumor cells were plated on the basolateral side of the insert membrane facing the lower chamber and loaded with gp33 peptide, while P14 CTLs were added to the upper chamber. This way, T cells were allowed to be in a very close proximity to tumor cells but were expected to migrate only through the 12 μm membrane, and thus directly interact with tumor cells, while EVs could unrestrictedly travel through both types of membranes. We observed that the frequency of ZsG-positive T cells (**Figure 5E**) was significantly higher in 12 μm pore-size inserts in comparison to 0.4 μm ones, indicating that while EVs may contribute, cell-cell contact is the predominant mechanism of the observed intercellular transfer.

## Discussion

In the current study, we generated an *in vivo* mouse ZsG-expressing tumor model that allowed us to track intercellular transfer of cytoplasm from the tumor cells to TILs based on fluorescent signal. Unexpectedly, we found that although many immune cells participated in such transfer, its intensity differed between cell types. Myeloid lineage cells demonstrated the highest level of ZsG uptake and subcellular localization of fluorescence appeared to be primarily punctate. Both observations are consistent with the phagocytic nature of these cells. In this regard, plasmacytoid DCs, that are overall considered to be less phagocytic (44), had the least amount of fluorescence. Most surprisingly, lymphoid cells also acquired ZsG fluorescence from tumor cells. Specifically, activated/differentiated T cells such as Tregs and various CTLs including exhausted T cells were the brightest among lymphoid subsets and had predominantly homogeneous distribution of fluorescence, suggesting that these T cells might be able to integrate tumor cytoplasm into their own.

One possibility of observed cytoplasmic transfer in the TME is through extracellular vesicles, such as exosomes, that are believed to be able to fuse with the plasma membrane of recipient cells and release their content (45–47). Alternatively, T cells may take in tumor cytoplasm from tumor cells by direct physical contact. Immune cells have been previously shown to engage in such cell-to-cell interactions. For example, some *in vitro* studies suggest that macrophages can form gap junctions between each other or with epithelial cells, thereby exchanging cytoplasm (48, 49). More recently, human lymphocytes (including T and B cells) were reported to exchange intracellular material through gap junctions in co-culture experiments using a dye transfer readout (50). The so-called cytoplasmic bridges (CBs), structures similar to nanotubes, were shown to be responsible for protein transfer between senescent fibroblasts and NK cells *in vitro* (51). In the same study, transfer of cytoplasm from pancreatic cells to NK cells and, importantly, to T cells was also demonstrated in an *in vivo* model of premalignant pancreas in K-Ras/mRFP mice; however, formation of CBs was not confirmed *in vivo*. It is well-known that exosomes circulate throughout the body via blood and lymph (52) and therefore it is not unreasonable to assume that they should easily travel from a tumor on one flank to a tumor on the opposite flank. Indeed, our results provide clear evidence of distant transfer, where P14 and OT-I CTLs isolated from non-fluorescent B16^OVA+^ tumors acquired ZsG fluorescence. However, the intensity of such transfer in these cells was reduced in comparison to the T cells isolated from B16^ZsG+GP33+^ tumors. Similarly, in our transwell assay, T cells that were allowed to directly interact with tumor cells exhibited more transfer than T cells that could only obtain ZsG protein from extracellular vesicles. Therefore, we think that physical interaction of cells in the TME may play a dominant role in the intercellular transfer and propose to call such type of transfer the “mosquito effect”.

Our scRNA-seq analysis of ZsG-negative and ZsG-positive T cells revealed striking differences between the two groups. First, the ZsG-positive group was enriched in Tregs and exhausted CD8 T cells. Second, comparison within individual T cell subsets showed that ZsG-positive T cells were more metabolically active, but at the same time demonstrated a suppressed immune response signature. These results raise an important, and perhaps the most interesting question, of whether acquisition of tumor material is simply a consequence of T cell activation or whether intercellular transfer has a functional impact on T cells. Increasing experimental evidence highlights the importance of cellular content exchange between tumors and the immune system, where tumors benefit while immune cells suffer detrimental consequences. For example, it has been demonstrated that low-avidity CTLs can capture peptide-major histocompatibility complexes from tumor cells via trogocytosis, decreasing Ag density on tumor cells and thereby making them poor targets for high-avidity CTLs (53). In a more recent study, a similar process was observed with chimeric antigen receptor (CAR) T cells, where trogocytosis-mediated Ag transfer from tumor cells to CAR T cells resulted not only in a poor cytolytic capacity towards tumor cells but also in fratricide among CAR T cells (54). Transfer of cellular material from macrophages to tumor cells through nanotubes in an *in vitro* pancreatic cancer model induced tumor cell reprogramming, resulting in increased tumorigenic potential (55). Furthermore, Saha et al. demonstrated that tumors can hijack mitochondria from T cells through nanotubes, thereby depleting immune cells of their energy source (56). Tumor-derived exosomes have also been implicated in shaping immune responses. For example, melanoma-produced exosomes were shown to promote formation of a metastatic niche by reprogramming bone marrow progenitors (57). Gargiulo et al. has recently reported that chronic lymphocytic leukemia-produced exosomes impair CD8 T cells and support leukemia progression (58). Finally, myeloid-derived suppressor cells have been shown to transfer the metabolite methylglyoxal to CD8 T cells, compromising their effector function (59). Therefore, it is tempting to speculate that tumors may use intercellular transfer to shut down immune cells and evade the immune response.

In conclusion, we demonstrated in an *in vivo* mouse model that all tumor infiltrating cells, including CD4 and CD8 T cells, acquire cytoplasmic material from tumor cells. We also showed that T cells that received tumor cytoplasm differ from those that did not with respect to their activation and immune status. The molecular mechanism that T cells use for intercellular transfer and the functional impact of this “mosquito effect” remain to be identified. In addition, our findings open broader questions, such as: Is uptake of cytoplasm from other cells a general property of T cells? What exactly is being transferred? Finally, does intercellular transfer happen at steady state, and if yes, what are the functional consequences for participating cells?

## Methods

### Animals

C57BL/6J (stock no. 000664), B6.Cg-*Rag2^tm1.1Cgn^*/J (stock no. 008449) and C57BL/6-*Foxp3^tm1Flv^*/J (stock no. 008374) mice were obtained from the Jackson Laboratory. B6;D2-Tg(TcrLCMV)327Sdz/JDvs/J (P14) mice were a generous gift from Alfred Singer (NCI, NIH). All breedings were maintained at the University of Massachusetts, Amherst. This study was performed in accordance with the recommendations in the Guide for the Care and Use of Laboratory Animals of the National Institutes of Health. All animals were handled according to approved institutional animal care and use committee (IACUC) protocols of the University of Massachusetts.

### Doxycycline-mediated induction of ZsG transgene expression

Experimental mice, including control animals, were fed with 2 mg/mL doxycycline in drinking water supplemented with 10 mg/mL sucrose for four days prior to harvesting tumors. Doxycycline drinking water was replaced every other day. *In vitro*, tumor cell lines were cultured with 2 μg/mL doxycycline in culture media.

### Flow cytometry analysis

Flow cytometry data were acquired on BD LSR Fortessa or Cytek™ AURORA. The following monoclonal antibodies from BioLegend were used: CD8α (53-6.7), CD4 (RM4-5), PD1-bio (29F.1A12), Tim-3 (RMT3-23), CD45 (30-F11), TCRβ (H57-597), CD11b (M1/70), Ly6c (HK1.4), Ly6g (1A8), CD3 (145-2C11), NK1.1 (PK136), B220 (RA3-6B2), F480 (BM8), SiglecH (551), CD11c (N418), CD24 (M1/69), CD172 (P84), Vβ5.1-5.2(MR9-4), Vβ8.1-8.2 (KJ16-133.18), Helios (22F6), FoxP3 (FJK-16s), CD45.1 (A20), CD45.2 (104), and Streptavidin- AF647. MHCII (M5/114.15.2) was from eBioscience, T-bet (O4-46) was from BD Pharmingen.

Live cells were treated with anti-CD16/32 Fc block (2.4G2, BD Pharmingen) prior to staining with antibodies against surface markers. Staining for surface proteins was performed at 4 °C for 40 min, and FACS buffer (PBS + 0.5% BSA + 0.01% sodium azide) was used for washes.

For transcription factor staining, Foxp3/Transcription factor staining buffer set (eBioscience) was used, and staining was performed according to the manufacturer’s instructions.

Data from BD LSR Fortessa and Cytek™ AURORA were analyzed in FlowJo™ v10.9.0 Software. Gating strategies used in the analysis are included in the supplementary material (**Supplementary Figure 5**). Data from Cytek™ AURORA were gated on live CD45+ cells and exported to csv files as channel values with all compensated parameters. The data in the exported files were converted to 10x file formats with custom code, such that the experiments can be analyzed by the Seurat package (60) using R software version 4.2.1. Files for B16/MC38 TILs or B16^ZsG^/MC38^ZsG^ TILs were loaded as Seurat objects with all cells and all features, then merged into one object. Fluorescence intensity of the FITC channel (for ZsG) was added to the metadata and excluded as a variable for clustering. Data were scaled, then 15 features were used for PCA and UMAP analyses. Clusters were annotated based on the fluorescence level of cell type markers.

### T cell culture

T cells were cultured in RPMI supplemented with 10% fetal bovine serum, 1% HEPES, 1% sodium pyruvate, 1% penicillin/streptomycin, 1% L-glutamine, 1% non-essential amino acids and 0.3% β-mercaptoethanol. Lymph nodes and spleens were harvested and gently tweezed to remove lymphocytes. Spleen cells were then lysed using ACK lysing buffer (KD Medical) to remove erythrocytes. Lymph node T cells were enriched for via antibody-mediated depletion of B cells using anti-mouse IgG magnetic beads (BioMag, Qiagen). For pure CD8 or CD4 T cells, CD4 or CD8 T cells were removed via anti-rat IgG magnetic beads (BioMag, Qiagen) following incubation with anti-mouse CD4 (GK1.5) or CD8 (2.43) antibodies conjugated with rat IgG, respectively.

For 24-hour activation, P14 T cells were stimulated with plate-bound anti-CD3 mAbs (1 μg/mL) and anti-CD28 mAbs (5 μg/mL). For CTL generation, P14, OT-I or polyclonal CD8 T cells were stimulated with irradiated splenocytes and soluble anti-CD3 mAbs (2 μg/mL). 48h after activation IL-2 was added to culture media (100 U/mL) and cells were differentiated for additional 3 days. For activation of CD4 T cells, polyclonal CD4 T cells were stimulated with irradiated splenocytes and soluble anti-CD3 mAbs (2 μg/mL) and IL-2 (200 U/mL). From 48h after activation, cells were differentiated for an additional 3 days in culture media with IL-2 (200 U/mL).

### Cloning, lentiviral vectors and transduction

The ZsGreen gene was subcloned into the pENTR/D-TOPO vector (Life technologies) and then inserted into the pInducer21 vector (Addgene, #46948) by Gateway technology. Gp33 minigene was cloned into pHRST-IRES-eGFP lentiviral vector (Harvard Medical School plasmid repository). Lentiviruses were produced from the co-transfection of HEK-293T cells with empty or ZsGreen-expressing pInducer21 vector and packaging plasmids pVSVg (Addgene, #8454) and psPAX2 (Addgene, #12260) at the ratio of 4:3:1 using Lipofectamine 2000 (Life Technologies) according to manufacturer’s protocol. Viral supernatants were collected 24 hours after transfection, filtered and used immediately for transduction. B16-F10 or MC38 cells plated in 6-well plates on the previous day were spinfected (900 RPM, 90 minutes, 37°C) with virus and polybrene (4 μg/mL).

### Tumor cell lines

B16-F10 melanoma cells were obtained from ATCC. MC38 and B16^Ova^ cell lines were a gift from Dr. Rodriguez (Moffit Cancer Center). B16-F10 cells were cultured in DMEM supplemented with 10% FBS, and MCA-38 cells were cultured in RPMI supplemented with 10% FBS. To generate B16^ZsG^ and MC38^ZsG^, B16-F10 and MC38 were transduced with a pInducer21 lentiviral vector modified to express ZsGreen and FACS-sorted for GFP-positive population. To generate B16^ZsG+gp33+^, B16-F10 cells were first transduced with a pHRST-IRES-eGFP lentiviral vector modified to express gp33 peptide, and FACS-sorted for GFP-positive population, and then transduced with a pInducer21 lentiviral vector modified to express ZsGreen and FACS-sorted for ZsGreen-positive cells after induction with doxycycline.

To obtain confocal images, live tumor cells were incubated with doxycycline to induce ZsGreen expression, co-stained with 4’,6-diamidino-2-phenylindole (DAPI) and imaged using A1R-TIRF confocal microscope (Nikon).

### Peptide loading and co-culture experiments

B16^ZsG^ tumor cells (0.15 mln/well) were cultured overnight in a 24-well plate in the presence of IFNγ (10 μg/mL final concentration) to induce MHC class I expression and dox (2 μg/mL) to induce ZsG. On the next day, the gp33 peptide (1 μM final concentration) was added to the monolayer of tumor cells for 1 hour, then carefully washed away with PBS. P14+ T cells (0.1 mln/well) were added on top of tumor cells and cocultured with dox. 24 hours later, T cells were collected and analyzed by flow cytometry.

### Transwell assay

Polycarbonate cell culture inserts (Millipore Sigma) with pore size of 0.4 μm and 12 μm were used in the assay. The basolateral side of the inserts were coated with fibronectin and laminin (Sigma, 5 μg/ml final concentration each) for 1 hour, then washed with PBS. B16^ZsG^ cells were plated onto the coated membrane in the presence of IFNγ and dox in the media. On the next day, tumor cells were loaded with the gp33 peptide. Inserts were placed in a 24-well plate such that tumor cells were faced down towards the lower chamber, and P14+ CTLs were added to the upper chamber. 24 hours later, T cells were collected from the upper chamber of 0.4 μm inserts, and from both the lower chamber and basolateral side of 12 μm inserts, then analyzed by flow cytometry.

### Tumor experiments

Mice were injected subcutaneously in the right flank (or both flanks) with 2.5 x 10^5^ tumor cells. For studies involving adoptive T cell transfer, 2 x10^6^ P14 or OT-I CTLs were injected intravenous into mice once tumors were established (at day 8 after tumor inoculation). For TIL isolation, tumors were minced and digested with 1 mg/mL collagenase D and 200 μg/mL DNase mix (Roche) by incubating at 37°C for 30 min with mixing every 5 min. The digestants were then passed through a 40-micron filter, spun at 1250 rpm for 5 min, and resuspended in FACS buffer.

### Single-cell RNA sequencing

Cells were stained and sorted on ZsG-positive and ZsG-negative cells using a BD FACSAria Fusion instrument (BD Biosciences). After sorting, cells were counted using a Cellometer K2 cell counter (Nexcelom Bioscience) and by manual counting via hemocytometer. Single cell gene expression profiling was performed using the Chromium Next GEM Single Cell 3′ v3.1 (Dual Index) kit. Each cell suspension was loaded onto a well of Chip G on the 10x Genomics Chromium Controller System following the manufacturer’s user manual (10x Genomics). Barcoding and cDNA synthesis were performed according to the manufacturer’s instructions. Qualitative analysis of cDNA was performed using the 2100 Agilent Bioanalyzer High Sensitivity assay. The cDNA libraries were constructed using the 10x Chromium Single cell 3’ Library Kit v3.1 (dual index) according to the manufacturer’s protocol. Quality assessment of final libraries was done on Qubit fluorometer using a DNA High Sensitivity assay (Thermo Scientific) and a 2100 Agilent Bioanalyzer High Sensitivity assay (Agilent Technologies). Libraries were sequenced on an Illumina NextSeq 500 using the NextSeq 500/550 Mid Output Kit v2.5 (150 Cycles) sequencing kit, with the following read length: 28 bp Read1 for the 10x cell barcode and UMI, 90 bp Read 2 for the insert, and 10 bp I7 and I5 for the sample index. Phix (Illumina) was spiked in at 1% as per kit manual recommendation (10x Genomics).

The 10x Could Analysis Cell Ranger Pipeline (Cellranger version 6.1.2) was used to align reads and generate feature-barcode matrices. Reads were aligned to *Mus musculus reference genome* (Mouse mm10 v3.0.0). The *aggr* pipeline was used to combine data from multiple samples into an experiment-wide feature-barcode matrix and analysis. The 10x Genomics Loupe Browser was used for visualization, initial quality assessment, and filtering of single cell gene expression data. Single Cell Gene Expression was performed at the Genomics Resource Laboratory, University of Massachusetts Amherst, MA.

### Analysis of scRNA-seq

Data analyses were performed using the Seurat package (60) in the R software version 4.2.1. For the dataset of sorted CD45+ ZsG-negative and ZsG-positive TILs, the Seurat object was first generated by keeping all genes expressed by at least 3 cells. Cells were kept if they contained between 1000 to 6000 unique features and less than 10% mitochondrial genes. Data were normalized, scaled, then the top 2000 variable genes were used for the Principal Component Analysis (PCA) and generation of Uniform Manifold Approximation and Projection (UMAP) plots. Cluster identities were inferred by analyzing the distribution of known cell type marker genes, and by calculating the top marker genes for each cluster. Annotation of Dendritic cells for DC1/DC2/pDCs were done by re-clustering the subset, applying the same Seurat analysis pipeline, then analyzing the distribution of cell type marker genes. The Monocyte/Macrophage cells were re-clustered with the same Seurat analysis pipeline. Signature plots of Monocyte/Macrophage cells were generated by calculating the average expression level of marker genes per cell.

Analyses for the data set of sorted CD45+CD3+ ZsG-negative and ZsG-positive TILs were performed using the ProjecTILs (42) and Seurat packages for the R software version 4.2.1. The Seurat object was first generated using the read.sc.query function by keeping all genes expressed by at least 3 cells and all cells with at least 50 unique features. The cells were filtered for those with less than 6000 unique features and less than 15% mitochondrial genes. To ensure our dataset only included T cells, the percentage of reads aligned to CD3 genes (*Cd3d, Cd3e, Cd3g*) among all reads in a cell were calculated, and cells with less than 0.055% CD3 genes were removed. Cell types were annotated using the make.projection and cellstate.predict functions with the reference dataset from the ProjecTILs package; 1390 cells were retained for further analyses. Expression of select genes for the annotated cells were visualized by dot plots or violin plots. Pie charts and bar plots for cell type counts were generated with the GraphPad Prism software. Differentially expressed genes were calculated by the Wilcoxon test between the ZsG- cells and ZsG+ cells using the find.discriminant.genes function.

### Pathway analyses (gene ontology and GSEA)

Differentially expressed genes (DEGs) between all ZsG-positive TILs or all ZsG-negative TILs were filtered for upregulated (Log_2_-fold change > 0, p-value <0.05) or downregulated (Log_2_-fold change < 0, p-value <0.05) genes, then submitted to Metascape (61) for gene ontology analysis. DEGs between ZsG-positive and ZsG-negative cells within each cell type were ranked by the metric: -Log_10_(p-value) * (Log_2_FC direction). Preranked GSEA was performed using the GSEA software (62, 63) for each cell type using mouse ortholog Hallmark gene sets.

### Statistical analysis

Statistical analyses, including tests for normality of sample groups and comparative tests were performed with Prism 9 (GraphPad software). *P*-values were determined using unpaired Student’s t-test, Mann-Whitney U test and Wilcoxon test as indicated in the figure legends. All statistical tests are two-tailed.

## Data availability statement

The datasets presented in this study can be found in online repositories. The names of the repository/repositories and accession number(s) can be found here: GSE246498 (GEO).

## Ethics statement

The animal study was approved by IACUC of University of Massachusetts, Amherst. The study was conducted in accordance with the local legislation and institutional requirements.

## Author contributions

KH: Data curation, Formal analysis, Investigation, Methodology, Writing – review & editing, Software, Validation, Visualization. DR: Formal analysis, Investigation, Writing – review & editing. IT: Formal analysis, Investigation, Visualization, Writing – review & editing. AL: Investigation, Visualization, Writing – review & editing. LP: Investigation, Writing – review & editing, Conceptualization, Data curation, Formal analysis, Funding acquisition, Methodology, Project administration, Supervision. EP: Conceptualization, Data curation, Formal analysis, Investigation, Methodology, Project administration, Supervision, Validation, Writing – original draft, Writing – review & editing.

## Supplementary material

Supplementary Figure 1
**(A)** Flow cytometry analysis of the expression of ZsG in B16^ZsG^ clone before (grey) and after (green) addition of dox. **(B)** Confocal microscopy image of B16^ZsG^ cells treated with dox.

Supplementary Figure 2
**(A)** Bubble plot showing key marker expression in TIL populations from B16^ZsG^ tumors. **(B)** Heatmap showing the top 10 characteristic genes for each monocyte/macrophage cluster. **(C)** UMAP plots of selected monocyte and macrophage markers in re-clustered monocytes/macrophages. **(D)** Cx3cr1 expression in re-clustered monocytes/macrophages.

Supplementary Figure 3
**(A)** Violin plots showing expression of key surface markers in TIL populations from B16 and B16^ZsG^ tumors **(B)** UMAP visualization of flow cytometry data (left) and ZsG intensity (right)in TILs collected from MC38 and MC38^ZsG^ tumors. **(C)** ZsG MFI in CD4 and CD8 TILs collected from MC38^ZsG^ tumors presented as a fold change relative to TILs from MC38 tumors. (**D**) Summary graph of ZsG MFI in intratumorally injected “spike” CD4 T cells in comparison to endogenous CD4 TILs from B16^ZsG^ tumors. Mean ± s.e.m, Mann-Whitney U test between MC38 TILs and MC38^ZsG^ TILs and Wilcoxon test between CD4 and CD8 MC38^ZsG^ TILs **(C)**, Mann-Whitney U test **(D)**: **p<0.01, ***p<0.001, ****p<0.001. Data represent three **(A, B)** and 2 **(D)** independent experiments or are pooled from multiple independent experiments **(C)**.

Supplementary Figure 4
**(A)** Reference map of TIL transcriptomic states from Andreatta et al (41) and a pie chart showing proportions of each TIL subset. **(B)** Bubble plot showing marker gene expression in TIL subsets (T cell scRNA-seq data).

Supplementary Figure 5
**(A, B)** Gating strategy for FACS analyses. Figures with the gating strategy used are indicated by arrows. **(A)** Gating strategy for TILs from *in vivo* experiments. **(B)** Gating strategy for CD8 T cells cocultured with B16^ZsG^ tumor cells. For all experiments, cells were identified by single cell gating on FSC-A/FSC-H before acquisition and only singlets were acquired.
