# Supplementary material for: The mosquito effect: regulatory and effector T cells acquire cytoplasmic material from tumor cells through intercellular transfer
Source: Front Immunol. 2023 Dec 20;14:1272918. doi: 10.3389/fimmu.2023.1272918 (PMC10765531; doi:10.3389/fimmu.2023.1272918)

Supplemental figure 1

A

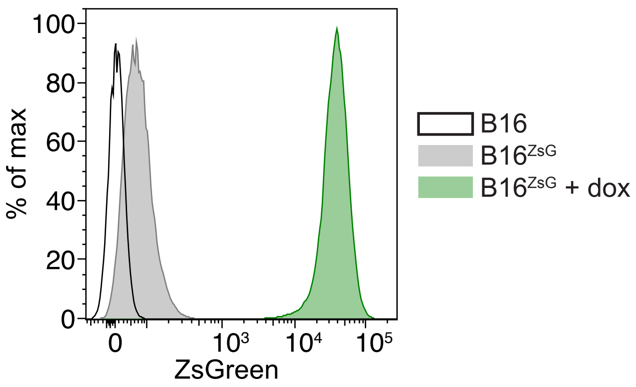

B

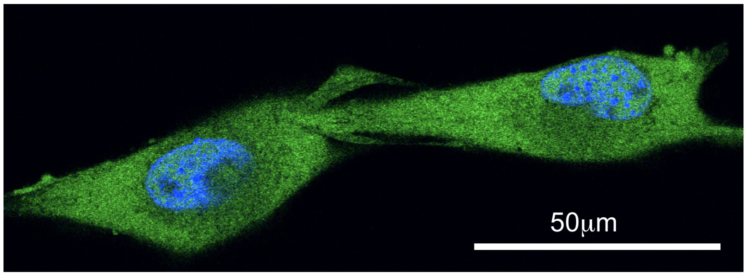

Supplemental figure 2

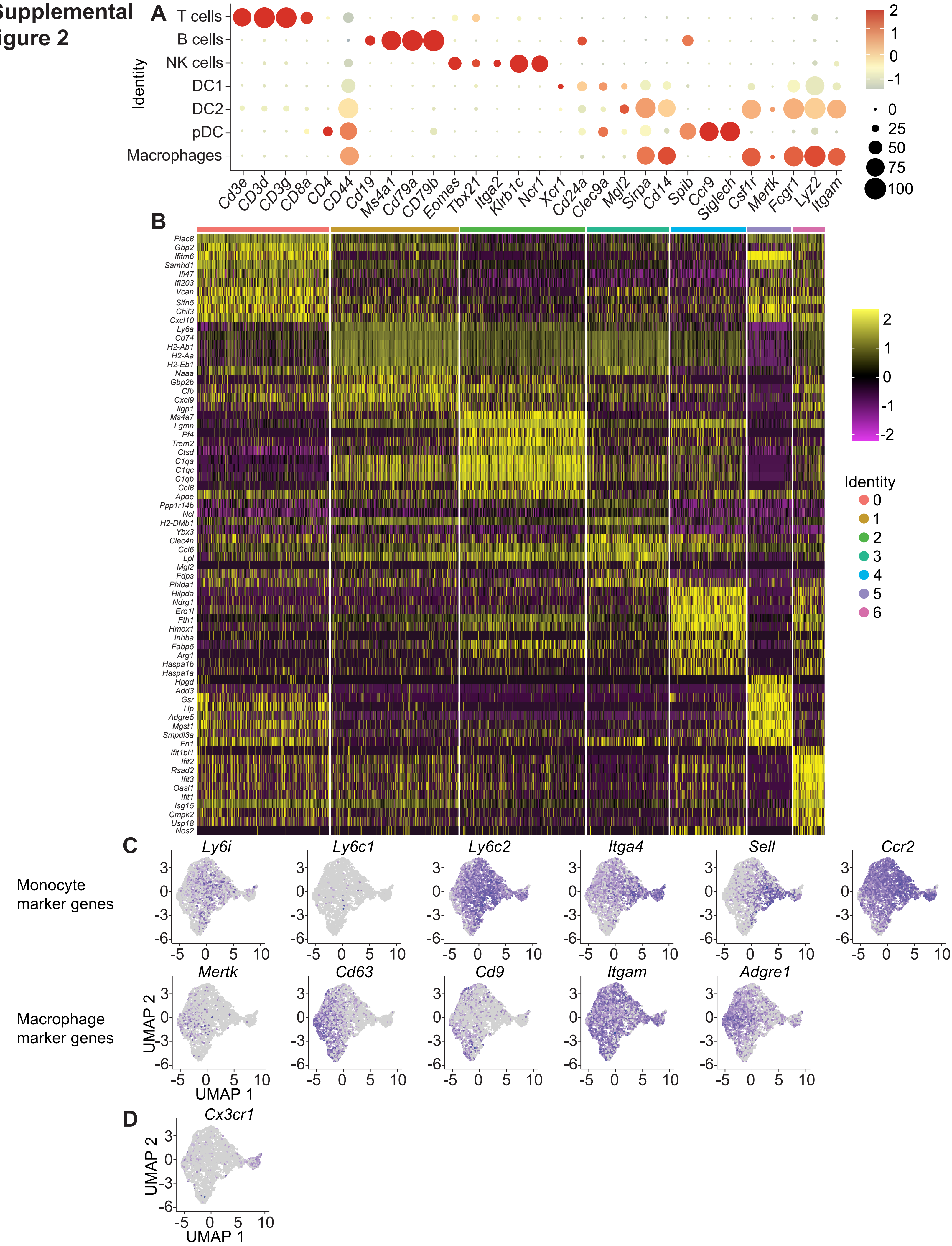

Supplemental figure 3

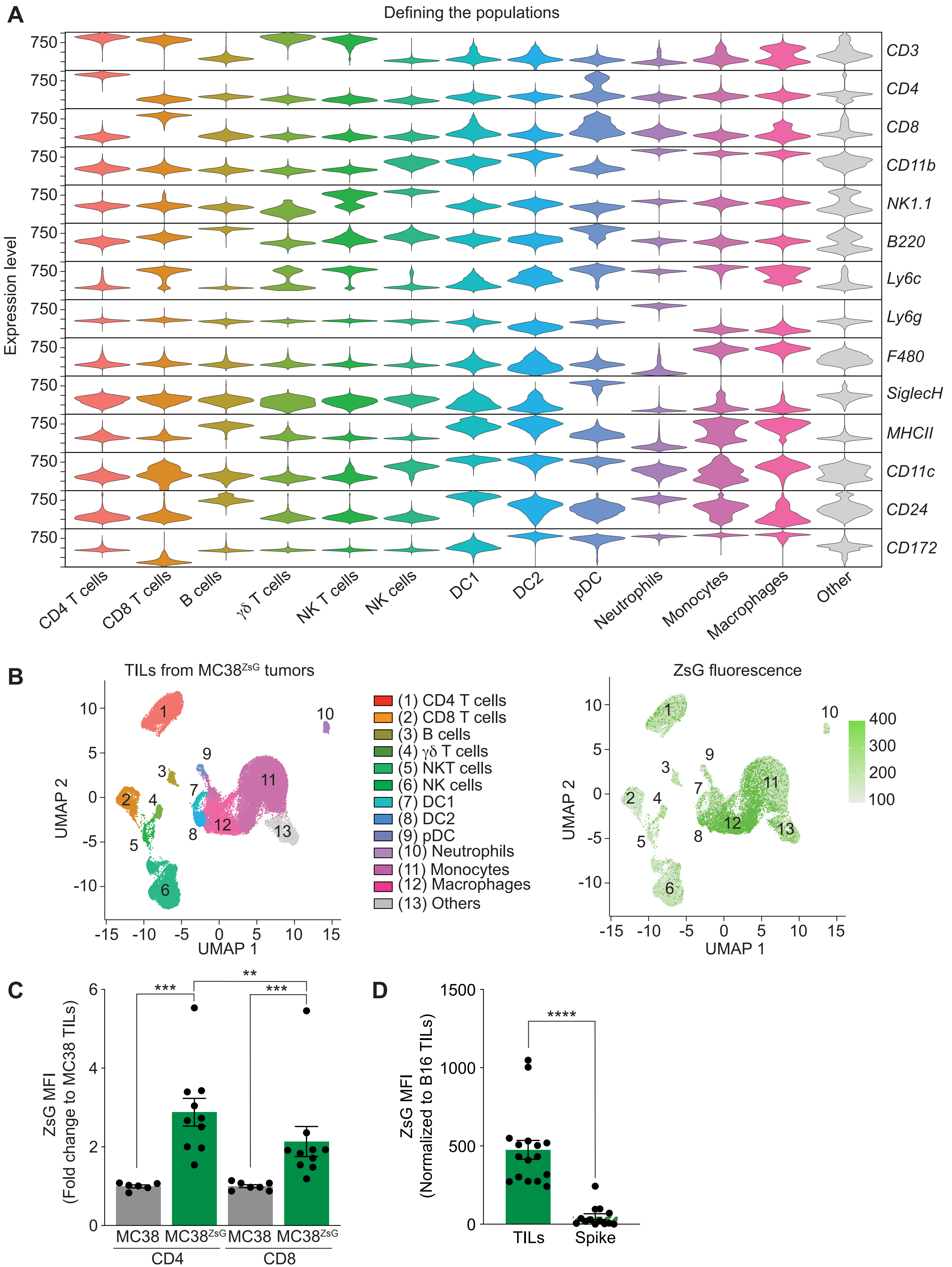

Supplemental figure 4

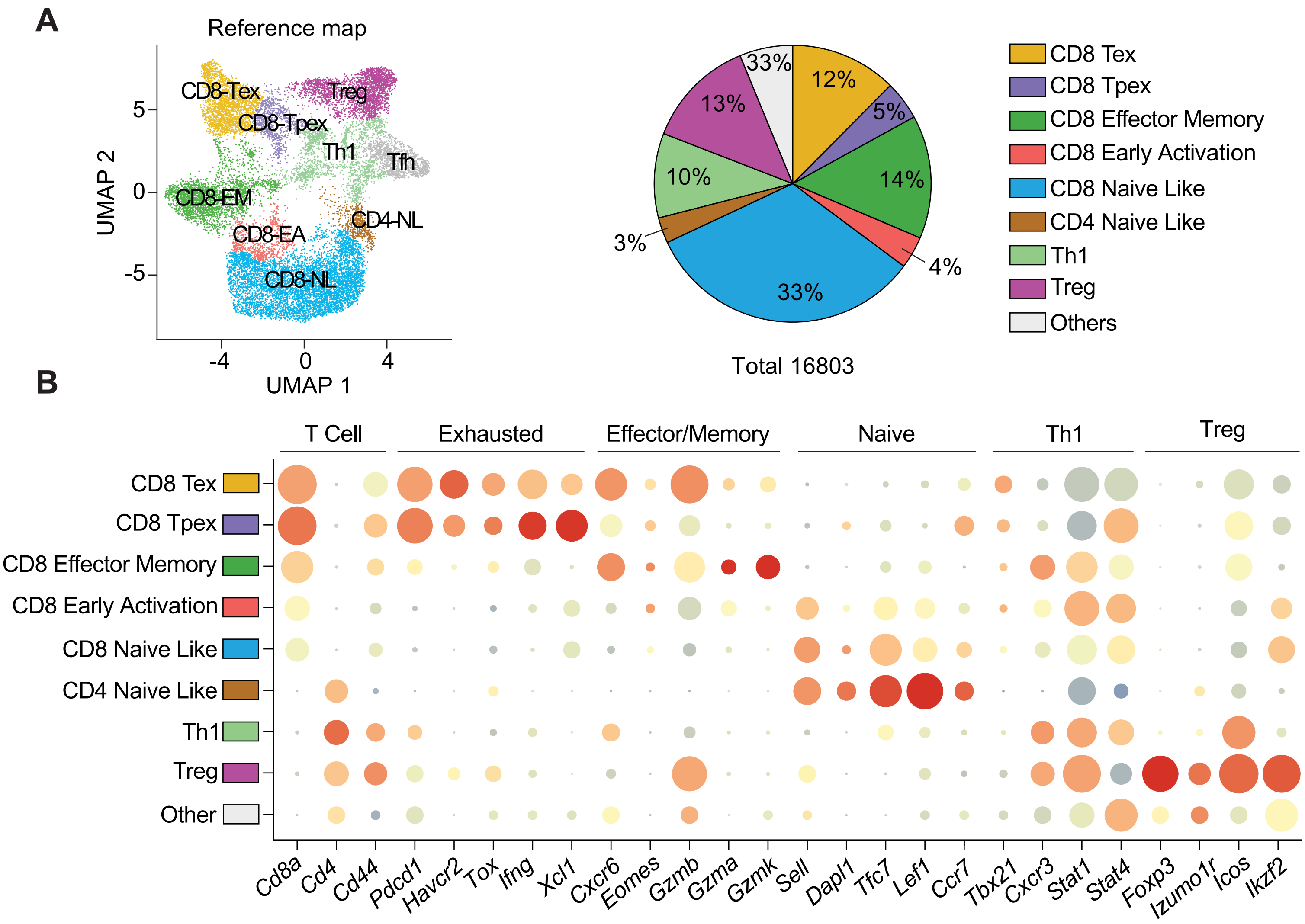

Supplemental figure 5

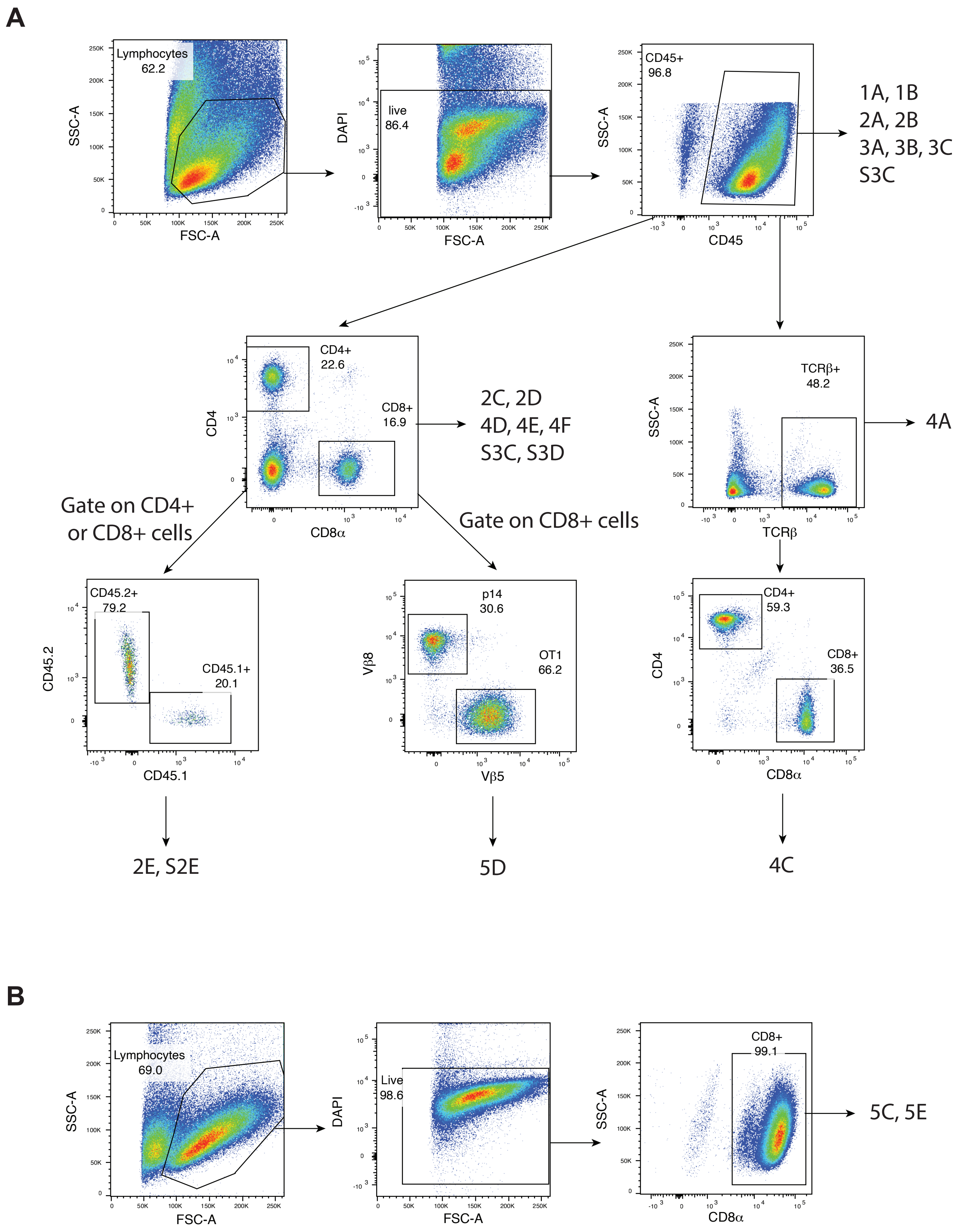

Supplement: Supplementary Figure 1 — (A) Flow cytometry analysis of the expression of ZsG in B16ZsG clone before (grey) and after (green) addition of dox. (B) Confocal microscopy image of B16ZsG cells treated with dox. [file DataSheet_1.pdf]
